# Sleeping quality, sleeping habits, and their association with BMI in children in southern China

**DOI:** 10.3389/fped.2025.1481263

**Published:** 2025-06-24

**Authors:** Chuican Huang, Yue Lei, Weijia Wu, Qing Luo, Wenting Cao, Junwei Xie, Hongai Li, Lichun Fan

**Affiliations:** ^1^ Clinical Nutrition Department, Hainan Women and Children’s Medical Center, Haikou, China; ^2^ School of Medicine, Zhejiang University, Zhejiang, China; ^3^ Department of Epidemiology, International School of Public Health and One Health, Hainan Medical University, Haikou, China; ^4^ Department of Child Health Care, Hainan Women and Children’s Medical Center, Haikou, China

**Keywords:** CSHQ, sleep quality (SQ), sleep duration, bedtime, BMI, children

## Abstract

**Objectives:**

This study analyzes the effects of sleep quality along with sleep duration and bedtime on BMI in children aged 3–12 years and explores their role in the occurrence and development of obesity in children.

**Methods:**

This study conducted a cross-section study on children in 18 cities and counties of Hainan Province. The Children's Sleep Habits Questionnaire (CSHQ), along with a parents-report children's features survey, was used to assess children's sleep quality and habits, among other factors.

**Results:**

The multivariable analysis results showed that bedtime was positively correlated with childhood overweight or obesity. However, the relationship between the outcome and sleep duration in children remains uncertain. The results of the additive interaction model indicated that sleep duration between 9 and 11 h or 11 h and above, combined with poor sleep quality or earlier bedtime (before 21:00), served as protective factors against children's overweight or obesity. Conversely, children with adequate sleep duration but later bedtime had a higher risk of being overweight or obese [OR (odds ratio): 1.214, 95% CI: 1.069–1.379]. Children with sleep duration less than 9 h, regardless of bedtime, had an increased risk of adverse outcomes (OR: 1.394, 95% CI: 1.022–1.901; OR: 1.268, 95% CI: 1.039–1.548).

**Conclusion:**

Short sleep duration (<9 h) and late bedtime (>22:00) independently and synergistically increase obesity risk, while adherence to recommended sleep patterns (9–11 h, bedtime before 21:00) offers protection. Non-linear analyses confirm a heightened obesity risk below 9 h of sleep, with partial attenuation beyond 11 h. Poor sleep quality paradoxically associates with lower obesity risk.

## Introduction

Childhood obesity and sleep disorders have become a global public health priority over the past decade (1, 2). Studies indicate that insufficient sleep among children is prevalent worldwide, with regional variations. For instance, 13.3% of children aged 6–12 years in mainland China experience sleep deprivation on school days, 40% of preschoolers in Hong Kong, China, fail to meet recommended sleep duration, and 23.6% of children aged 1–14 years in Spain exhibit short sleep duration (3–6). Notably, data from the past decade reveal a parallel and prospectively associated trend between rising childhood obesity rates and declining average sleep duration (7, 8). As of 2016, WHO data revealed that approximately 340 million children and adolescents worldwide were affected by overweight or obesity (9). Concurrently, China’s 2020 national survey reported overweight and obesity rates of 11.1% and 7.9%, respectively, among children aged 6–17 years (10), with a persistently escalating trend observed in low-income countries (11).

Within growth trajectories, early-life exposure to obesogenic environments is critical for the development of obesity (12). In addition to common influencing factors, other unhealthy behaviors, such as poor sleep quality, insufficient sleep duration (13), reduced physical activity (14), and increased screen time (15), are associated with an elevated risk of weight gain and may have long-term negative consequences (11, 16, 17). The longitudinal study demonstrated that poor sleep habits at the age of 1 significantly increase the risk of being overweight at the age of 8 (18). Moreover, suboptimal sleep quality may exacerbate obesity by disrupting energy metabolism homeostasis (19). However, existing research findings remain inconsistent: while some studies have found a significant negative correlation between sleep duration and obesity (20), others have not observed a significant association, possibly due to sample size limitations or low reporting rates of sleep problems (21). Furthermore, the interaction between sleep-related variables—such as duration, quality, and bedtime—remains unclear, particularly in the context of Chinese children, on whom systematic evidence is lacking (22).

International health organizations have issued age-specific sleep recommendations for children, reflecting variations in sleep requirements across developmental stages: The 2019 WHO guidelines suggest that children under the age of 5 require 10–13 h of high-quality sleep per day (23), while the American Academy of Sleep Medicine recommends 9–12 h of sleep per day for children aged 6–12 y (24). In China, the Ministry of Education mandates that primary school students sleep no less than 10 h per night and go to bed no later than 21:20. However, in reality, children often face multidimensional sleep issues, such as having good sleep quality but insufficient duration or experiencing delayed bedtimes. The synergistic effects of these factors on weight-related indicators have yet to be elucidated.

This study, based on children from southern China, aims to address the following scientific questions: (1) Utilizing the Children’s Sleep Habits Questionnaire (CSHQ), along with a parent-reported children’s characteristics survey, we will assess whether sleep quality, duration, and bedtime independently or synergistically influence children’s weight development; (2) employing an additive model, we will quantify the interaction effects among sleep variables to provide evidence for the development of targeted intervention strategies. The findings of this study will fill gaps in the existing literature and serve as a reference for the evidence-based revision of children’s sleep health guidelines.

## Method

### Study design and population

The cross-sectional study is a part of the Major project of Hainan Province, a population-based project investigating the determinants of physical development in children aged 0–18 years across all 18 administrative divisions of Hainan Province (2019–2022). For the current analysis focusing on 3–12-year-olds (*n* = 44,768), we employed a multistage stratified cluster sampling design:

1. Primary sampling units (PSUs): 42 schools stratified by educational stage (kindergarten, primary school) and urbanization level (urban/rural).
2. Secondary sampling units (SSUs): Classrooms randomly selected within PSUs with probability proportional to student population size.

All eligible students within selected SSUs participated through parental consent (response rate: 92.1%). The protocol was approved by the Ethics Committee of Hainan Women and Children’s Medical Center (Ethics Committee approval number: 2021005), with written informed consent obtained from all caregivers.

According to previous literature, the rate of abnormal growth and development in infants and young children in South China is about 5.5%, and the rate of abnormal growth and development in adolescence is about 3.77%. Power calculations accounted for the clustered sampling design using the formula

$$n_{adjusted}=n_{simple}\times DEFF*$$

*: Design effect (DEFF) = 1.5 based on intraclass correlation coefficient (ICC = 0.05) from pilot data.

3–6 years: *n* = 6,109 [required *n* = 5,720 for α=0.05, power = 90% to detect OR (odds ratio) = 1.4].

6–12 years: *n* = 38,659 (required *n* = 35,700 for OR = 1.2 detection).

Actual samples exceeded requirements across all strata, ensuring adequate power for subgroup analyses.

## Measures and variables

### Outcome

#### BMI

The height and weight information of the participating children were directly derived from the kindergarten physical examination database, calculated BMI as weight(kg)/height(m)^2^, converted to BMI z-scores using WHO AnthroPlus v3.2.2.

### Exposure variables

#### Sleep quality

Assessed via Chinese version of CSHQ (Cronbach’s α = 0.83 in validation subsample). Caregivers were asked to complete the CSHQ along with a caregivers-report children’s features survey. Eight dimensions were covered in this CSHQ, namely, bedtime resistance; sleep onset delay; sleep duration; sleep anxiety; night awakenings; parasomnias; sleep disordered breathing; and daytime sleepiness. The total CSHQ score was obtained by adding up the scores of all eight dimensions. Children with a higher score tend to have poor sleep quality. In this study, according to the CSHQ criteria, a total score of more than 41 was defined as poor sleep quality (25).

#### Bedtime

Bedtime is defined as the time during the night when children go to bed falling asleep after lights out. Temporal information was obtained from the caregivers-report children’s features survey. It was obtained from the question, “At about what time in the evening does your child go to sleep?” The variable was classified into an ordinal categorical variable and a 1 = “<21:00”, 2 = “21:00–22:00”, and 3 = “>22:00” was used.

#### Sleep duration

Children’s sleep duration was calculated based on the caregivers-report children’s features survey. The question “What time does your child wake up in the morning?” and the answer of bedtime were used to calculate children’s sleep duration. This variable is divided into three categories and a 1 = “< 9 h”, 2 = “9–11 h”, and 3 = “>11 h” was used.

### Covariate

Previous research suggested that children’s weight development and individual sleep status are related to child-, family-, and community-level variables (26). Adjusting for a series of confounding influence factors serves to disambiguate the bias to outcomes and conclusions. Therefore, some control variables were included in hypothesis testing models detailed in the analysis plan. Child-level variables included age and gender, Han nationality, Li nationality, or others and the time spent outdoors. Family-level variables included maternal education level. Finally, community-level variables included children’s residence type.

### Statistical analysis

Analyses were conducted in SPSS 26.0 and Stata 17.0. A series of restricted cubic spline (RCS) models were estimated to observe the trends of BMI with sleep duration and bedtime in children aged 3–12 years. First, a basic demographic analysis of the population included in the study was performed. For qualitative data, the composition ratio was used for brief introduction, and the chi-square test or Fisher’s exact probability method was used for comparison between groups. The quantitative data were described in the format of mean ± standard deviation. Next, significant results in univariate analysis were selected and included in the binary logistic regression model by “forward stepwise regression.” Modeled continuous sleep variables using RCS with 4 knots were placed at empirical percentiles (5th, 35th, 65th, and 95th). Reference points were set at age-specific sleep recommendations. The analytical framework employed multivariable regression models incorporating three-way interaction terms to examine the combined effects of sleep duration, sleep quality, and bedtime on pediatric BMI.

## Result

### General demographic characteristics

Information regarding the study sample characteristics according to weight status is given in Table 1. Among the 19,047 participants, the average BMI z-score was 16.72 ± 2.90, and there were more boys (62.9%) with obesity than girls (37.1%). The average score of the CSHQ was 58.90 ± 19.87, and 22.4% of the children had good sleep quality. Irrespective of normal weight children or overweight children or obese children, the proportion of poor sleep quality was relatively high, which could reach more than 70%. A few children did not spend any outdoor time, and most of the participating children (65.6%) spent 1 h playing outdoors. Bedtime of the sample was concentrated in 21:00–22:00, accounting for 51.1%, and a minority (13.3%) of children went to bed after 22:00. About 90% of the children had Screen Exposure, mainly in about 1 h (76.2%).

**Table 1:** General demographic characteristics.

Variables	*n* (%) 19,047 (100%)	Weight status		*p*-value
		Normal	Overweight and obesity	
Gender				<0.001
Girls	9,465 (49.7)	8,086 (52.7)	1,379 (37.1)	
Boys	9,582 (50.3）	7,248 (47.3)	2,334 (62.9)	
Nationality				<0.001
Han nationality	14,221 (74.7)	11,321 (73.8)	2,900 (78.1)	
Li nationality	4,135 (21.7)	3,460 (22.6)	675 (18.2)	
Others	686 (3.6)	550 (3.6)	136 (3.7)	
BMI z-score	16.72 ± 2.90			<0.001
Age groups (years)				<0.001
3–6	3,406 (17.9)	2,914 (19.0)	492 (13.3)	
7–12	15,641 (82.1)	12,420 (81.0)	3,221 (86.7)	
Type of residence				<0.001
Urban	6,353 (33.4)	4,923 (32.1)	1,430 (38.5)	
Rural	12,694 (66.6)	10,411 (67.9)	2,283 (61.5)	
Maternal education				<0.001
Primary	1,640 (8.9)	1,388 (9.4)	252 (7.0)	
Intermediate	10,964 (59.6)	8,885 (60.1)	2,079 (57.7)	
Secondary	3,506 (19.1)	2,745 (18.6)	761 (21.1)	
College or above	2,291 (12.5)	1,778 (12.0)	513 (14.2)	
Outdoor activity (h)				0.461
0	533 (2.8)	425 (2.8)	108 (2.9)	
1	12,491 (65.6)	10,029 (65.4)	2,462 (66.3)	
2	6,019 (31.6)	4,876 (31.8)	1,143 (30.8)	
CSHQ score	58.90 ± 19.87			0.161
Sleep quality				<0.001
Good	4,258 (22.4)	3,328 (21.7)	930 (25.0)	
Bad	14,789 (77.6)	12,006 (78.3)	2,783 (75.0)	
Bedtime				<0.001
<21:00	6,770 (35.5)	5,589 (36.4)	1,181 (31.8)	
21:00–22:00	9,739 (51.1)	7,795 (50.8)	1,944 (52.4)	
>22:00	2,538 (13.3)	1,950 (12.7)	588 (15.8)	
Screen exposure (h/day)				0.085
0	1,902 (10.0)	1,521 (9.9)	381 (10.3)	
1	14,518 (76.2)	11,736 (76.5)	2,782 (74.9)	
2	2,625 (13.8)	2,075 (13.5)	550 (14.8)	

### Analysis of influencing factors of abnormal weight in children

Table 2 presents the results of both univariate and multivariate analyses combined. Models 1 and 2 present the outcomes of univariate analyses focused on demographic characteristics and influencing factor variables, respectively. Model 3 is the product of a multivariate analysis.

**Table 2:** The result of univariate and multivariate analysis of variables influencing children's weight outcome.^a^

Variables	Model 1			Model 2^b^			Model 3^c^		
	OR (95% CI)	X^2^	*p*-value	OR (95% CI)	X^2^	*p*-value	OR (95% CI)	X^2^	*p*-value
Nationality									
Han nationality	Ref.			—	—	—	—	—	—
Li nationality	0.762 (0.695–0.835)	33.63	<0.001	—	—	—	—	—	—
Others	0.965 (0.797–1.170)	0.13	0.965	—	—	—	—	—	—
Type of residence									
Rural	Ref.			—	—	—	—	—	—
Urban	1.325 (1.230–1.427)	55.02	<0.001	—	—	—	—	—	—
Yearly household income (RMB)									
0–30,000	Ref.			—	—	—	—	—	—
30,000–50,000	1.189 (1.086–1.302)	14.09	<0.001	—	—	—	—	—	—
50,000–100,000	1.405 (1.271–1.554)	44.22	<0.001	—	—	—	—	—	—
>100,000	1.413 (1.227–1.628)	22.96	<0.001	—	—	—	—	—	—
Maternal education level									
College or above	Ref.			—	—	—	—	—	—
Primary	0.629 (0.533–0.743)	29.80	<0.001	—	—	—	—	—	—
Intermediate	0.811 (0.929–0.905）	14.13	<0.001	—	—	—	—	—	—
Secondary	0.961 (0.846–1.091)	0.38	0.537	—	—	—	—	—	—
Outdoor activity (h)									
0	—	—	—	1.084 (0.869–1.352)	0.51	0.474	1.054 (0.843–1.318)	0.212	0.645
1	—	—	—	1.047 (0.969–1.132)	1.34	0.246	1.053 (0.972–1.140)	1.581	0.209
2	—	—	—	Ref.			Ref.		
Sleep quality									
Good	—	—	—	Ref.			Ref.		
Bad	—	—	—	0.829 (0.763–0.902)	19.22	<0.001	0.825 (0.759–0.889)	20.08	<0.001
Bedtime									
<21:00	—	—	—	0.847 (0.782–0.918)	16.46	<0.001	0.873 (0.804–0.949)	10.26	0.001
21:00–22:00	—	—	—	Ref.			Ref.		
>22:00	—	—	—	1.209 (1.089–1.343)	12.62	<0.001	1.186 (1.065–1.320)	9.67	0.002
Sleep duration (h)									
9–11	—	—	—	Ref.			Ref.		
<9	—	—	—	1.160 (1.022–1.317)	5.25	0.022	1.104 (0.969–1.257)	2.21	0.137
>11	—	—	—	0.876 (0.807–0.951)	9.97	0.002	0.937 (0.860–1.021)	2.20	0.137
Screen exposure (h/day)^d^									
0	—	—	—	1.057 (0.938–1.191)	0.82	0.366	1.043 (0.924–1.177)	0.46	0.499
1	—	—	—	Ref.			Ref.		
2	—	—	—	1.118 (1.009–1.239)	4.55	0.033	1.121 (1.010–1.245)	4.57	0.033

BAZ, BMI-for-age z-score.

^a^ BAZ ≥ 1 is considered overweight and BAZ ≥ 2 is considered obesity.

^b^ Model 2 adjusted by age, sex, nationality, and type of residence.

^c^ Model 3 adjusted by age, sex, nationality, and type of residence.

^d^ Screen exposure time includes watching TV, tablet, mobile phone, electronic games, etc.

The findings indicate that, among the demographic variables, children’s nationality (Li nationality, OR: 0.762, 95% CI: 0.695–0.835, *p* < 0.001), the type of residence (urban or rural) (urban group, OR: 1.325, 95% CI: 1.230–1.427, *p* < 0.001), maternal education level (primary group, OR: 0.629, 95% CI: 0.533–0.743, *p* < 0.001), and family economic situation (>100,000 RMB groups, OR: 1.413, 95% CI: 1.227–1.628, *p* < 0.001) are associated with the weight growth of children. In the other modifiable independent variables, which can be improved through interventions, statistical associations were identified between sleep quality, bedtime, screen exposure time, and childhood overweight or obesity. Notably, poor sleep quality showed a negative correlation with the outcome variable (OR: 0.825, 95% CI: 0.759–0.889, *p* < 0.001). In addition, bedtime after 22:00 (OR: 1.186, 95% CI: 1.065–1.320, *p* = 0.002) and daily screen exposure exceeding 2 h (OR: 1.121, 95% CI: 1.010–1.245, *p* = 0.033) emerged as risk factors for overweight or obesity in children. In the univariate analysis, a correlation was observed between children’s sleep duration and outcomes. Specifically, shorter sleep duration (<9 h) shows a significant positive association with overweight/obesity prevalence, whereas children who slept more than 11 h/day had a lower likelihood of developing overweight/obesity. However, upon inclusion of this independent variable in the multivariate model, its statistical association became non-significant (<9 h group, OR: 1.104, 95% CI: 0.969–1.257, *p* = 0.137; > 11 h group, OR: 0.937, 95% CI: 0.860–1.021, *p* = 0.137). There was no statistically significant association between time spent outdoors and overweight or obesity in children.

### Sleep duration and bedtime of children and their risk of developing overweight or obesity

To further explore the relationship between sleep duration and bedtime with the outcome variable, both of these independent variables were separately included as continuous variables in binary logistic regression models, with the models adjusted for covariates (Table 3). The results indicate that sleep duration is one of the protective factors for the outcome variable (OR: 0.958, 95% CI: 0.926–0.991, *p* = 0.014), showing a negative correlation with the outcome variable. As sleep duration increases, the risk of weight abnormalities decreases. Conversely, for the children in this study, a later bedtime is positively correlated with the outcome and is identified as one of the risk factors for overweight or obesity (OR: 1.071, 95% CI: 1.020–1.124, *p* = 0.006).

**Table 3:** Sleep duration and bedtime logistic regression analysis results related to weight development.

Variable	Model 1^a^		Model 2^b^		Model 3^c^	
	OR (95% CI)	*p*-value	OR (95% CI)	*p*-value	OR (95% CI)	*p*-value
Sleep duration	0.916 (0.886–0.946)	<0.001	0.940 (0.909–0.971)	<0.001	0.958 (0.926–0.991)	0.014
Bedtime	1.150 (1.097–1.205)	<0.001	1.131 (1.079–1.185)	<0.001	1.071 (1.020–1.124)	0.006

^a^ Model 1 adjusted for age and gender.

^b^ Model 2 adjusted for age, gender, ethnicity, and type of residence.

^c^ Model 3 adjusted for age, gender, ethnicity, type of residence, maternal educational level, and family economic status.

### Non-linear relationship analysis

**Figure 1:**
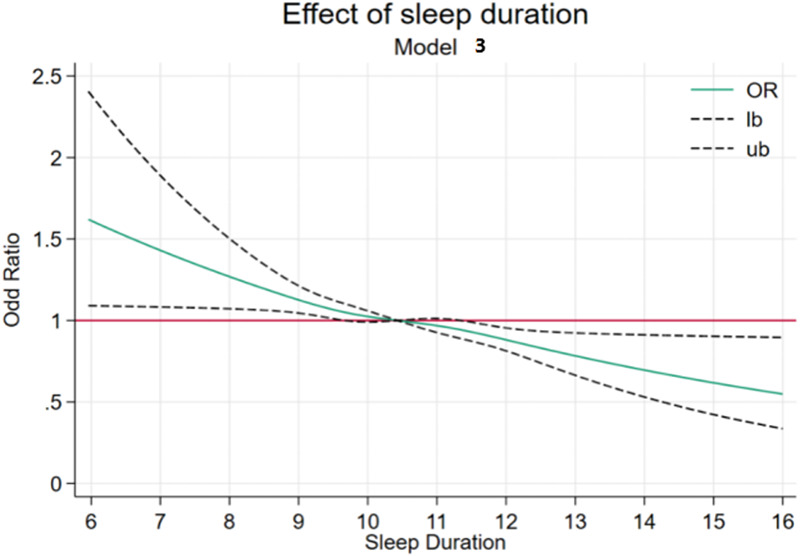
RCS plot of sleep duration.

**Figure 2:**
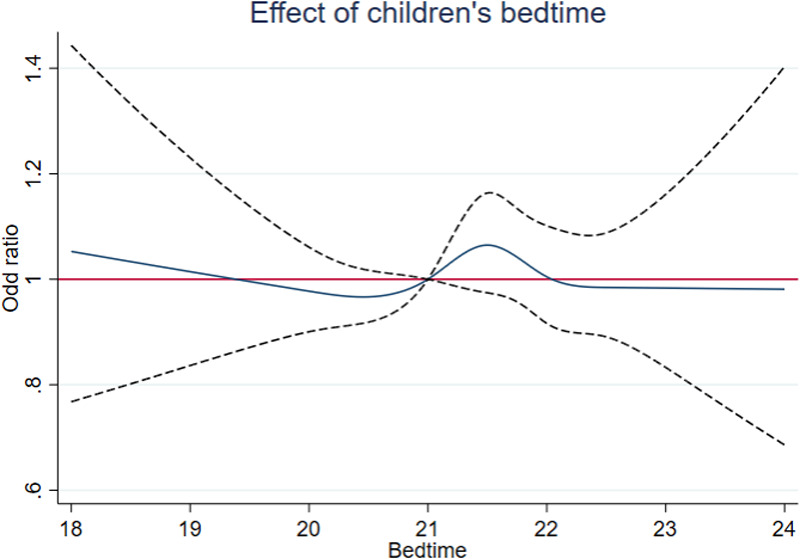
RCS plot of bedtime.

RCSs were implemented to model potential non-linear associations between sleep parameters (duration and bedtime) and BMI z-scores (Figures 1, 2). According to previous research, the number of nodes should be chosen between 3 and 7 (27). In the present study, for a more comprehensive data fitting, four nodes were selected based on the quartiles of the *x*-axis variable. For the analysis of the dose–response relationship between sleep duration and childhood overweight or obesity, four nodes were chosen at 8.73, 9.14, 11.14, and 12.38 (*P*5, *P*25, *P*75, and *P*95). For bedtime, four nodes were selected at 20:00, 21:00, 22:00, and 22:30 (*P*5, *P*25, *P*75, and *P*95).

As illustrated in Figure 1, we conducted RCS analysis using the recommended sleep duration of 10.5 h as the reference value to assess the dose–response relationship between varying sleep durations and overweight/obesity risk in children. Covariates including age, sex, residence, maternal education, and family socioeconomic status were adjusted in the models. The RCS curves revealed a significant non-linear association, with adjusted ORs at key inflection points as follows: 1.12 (1.02–1.23), 1.08 (1.02–1.13), 0.94 (0.86–1.01), and 0.80 (0.72–0.89). Notably, prolonged sleep duration demonstrated a negative correlation with the prevalence of weight abnormalities, indicating that the risk of overweight or obesity in children gradually decreased as sleep duration increased.

The RCS model results for bedtime in Figure 2 show that, using the relatively appropriate bedtime of 21:00 as a reference for analysis, the OR values (95% CI) for the four nodes are 0.98 (0.90–1.06), 1, 1.04 (0.91–1.10), and 0.98 (0.89–1.10). The curve between bedtime and the outcome exhibits fluctuations within a certain range but lacks statistical significance.

### Additive interaction effect analysis

Based on the multivariate regression and RCS findings identifying sleep quality, bedtime, and sleep duration as modifiable determinants of pediatric adiposity, the present study further analyzed whether there is an interaction among them, utilizing an additive interaction model for the analysis (Table 4).

**Table 4:** The interaction between sleep duration, bedtime, and sleep quality in relation to the weight development of children.

Variables		X^2^	*P*-value	OR (95%CI)	Protective factors	Risk factors
Sleep duration	Sleep quality				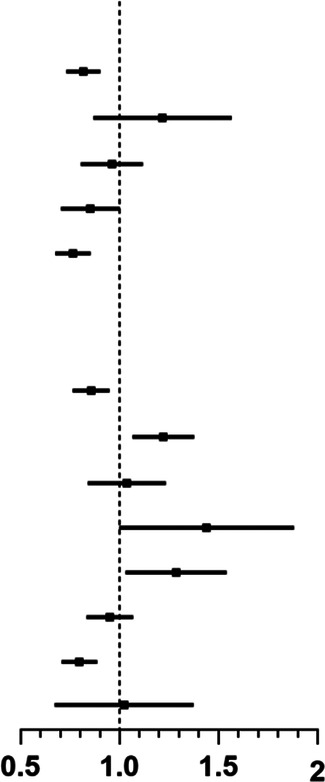	
9–11 h	Good	–	–	Ref.		
9–11 h	Bad	14.69	<0.001	0.814 (0.733–0.905)		
<9 h	Good	1.28	0.258	1.183 (0.885–1.581)		
<9 h	Bad	0.33	0.564	0.953 (0.809–1.122)		
>11 h	Good	3.59	0.058	0.844 (0.708–1.006)		
>11 h	Bad	19.75	<0.001	0.759 (0.678–0.857)		
Sleep duration	Bedtime					
9–11 h	21:00–22:00	–	–	Ref.		
9–11 h	<21:00	8.28	0.004	0.853 (0.765–0.951)		
9–11 h	>22:00	8.92	0.003	1.214 (1.069–1.379)		
<9 h	21:00–22:00	0.06	0.806	1.024 (0.846–1.240)		
<9 h	<21:00	4.39	0.036	1.394 (1.022–1.901)		
<9 h	>22:00	5.44	0.02	1.268 (1.039–1.548)		
>11 h	21:00–22:00	0.06	0.806	0.945 (0.835–1.07)		
>11 h	<21:00	16.17	<0.001	0.793 (0.709–0.888)		
>11 h	>22:00	0.01	0.925	0.983 (0.695–1.392)		

**Sleep duration, sleep quality, and their associations with overweight or obesity**: Using the age-specific recommended sleep duration (9–11 h) with optimal sleep quality (CSHQ ≤41) as the reference, the model demonstrated that moderate sleep duration (9–11 h), combined with poor sleep quality (OR = 0.814, 95% CI: 0.733–0.905), as well as extended sleep duration (>11 h) with poor sleep quality (OR = 0.759, 95% CI: 0.678–0.857), were both associated with a reduced risk of overweight or obesity in children. In contrast, short sleep duration (<9 h), combined with poor sleep quality, did not exhibit a statistically significant association.

**Sleep duration, bedtime, and their associations with overweight or obesity**: Using the recommended sleep duration (9–11 h) and normative bedtime (21:00–22:00) as the reference, the models identified distinct risk patterns. An earlier bedtime (before 21:00), in conjunction with moderate sleep duration (9–11 h) (OR = 0.853, 95% CI: 0.765–0.951) or extended sleep duration (>11 h) (OR = 0.793, 95% CI: 0.709–0.888), was associated with a lower risk of overweight or obesity. Conversely, a later bedtime (after 22:00), combined with moderate sleep duration, was linked to an increased risk of obesity (OR = 1.214, 95% CI: 1.069–1.379). Notably, short sleep duration (<9 h) consistently exhibited adverse effects regardless of bedtime: an earlier bedtime (<21:00) was associated with a 39% increased risk (OR = 1.394, 95% CI: 1.022–1.901), while a later bedtime (>22:00) was linked to a 27% elevated risk (OR = 1.268, 95% CI: 1.039–1.548). These findings suggest that in the context of insufficient sleep duration, the impact on overweight or obesity risk is primarily driven by sleep duration itself rather than modulated by bedtime.

## Discussion

Children, as a focal point of societal and familial concern, garner significant attention in their growth and development. Historically, children’s weight has been a primary focus of Early Childhood Development (ECD) (28), while in the past decade, children’s sleep hygiene has emerged as a hot topic in public health. The exploration of the relationship between sleep and children’s weight development is also a pressing issue awaiting clarification by researchers. This study focuses on the combined effects of sleep quality and sleep habits on children’s weight development.

Among the 19,047 children included in this study, the prevalence of overweight or obesity was higher in boys than in girls. Significant differences were observed between normal weight and overweight or obese children across age groups, urban–rural distribution, and maternal education levels. Preliminary analyses revealed marked disparities in sleep quality and bedtime among different weight groups for sleep-related independent variables, consistent with prior studies by Schultz et al. and Covington et al. (29–31). Further exploration of risk factors demonstrated that, in the adjusted multivariate model, later bedtime (after 22:00) and prolonged screen time (>2 h/day) were both associated with elevated risks of childhood overweight or obesity (OR = 1.186, 95% CI: 1.065–1.320; OR = 1.121, 95% CI: 1.010–1.245). However, suboptimal sleep quality exhibited a protective association with childhood overweight/obesity in this model (OR = 0.825, 95% CI: 0.759–0.889). Jebeile et al. noted in *The Lancet* that poor sleep quality may exacerbate energy regulatory dysfunction, potentially promoting weight gain (19). However, studies by Michels et al. and Cespedes et al. reported divergent findings, indicating no statistical association between sleep quality and obesity-related behaviors (32, 33). Although extensive research has explored mechanisms linking poor sleep quality to obesity, no definitive evidence currently establishes poor sleep quality as a causal factor for overweight or obesity.

Sleep duration and bedtime are two fundamental components of the sleep process and have been extensively examined in studies on children’s sleep hygiene. In the model presented in Table 3, sleep duration, treated as a continuous variable, demonstrated a statistically significant association with the outcome variable. It was identified as a protective factor against childhood overweight and obesity, with the risk of adverse outcomes decreasing as sleep duration increased. This finding aligns with the results of most existing studies (30, 32, 34). Conversely, bedtime demonstrated a statistically significant association in the model and was identified as a risk factor for overweight and obesity. A study by Anderson et al. on American children revealed that delayed bedtime was associated with elevated adolescent obesity prevalence. Specifically, compared with children maintaining early and regular bedtimes during preschool years, those with later bedtimes exhibited a multivariable-adjusted relative risk of 0.48 (95% CI: 0.29–0.82) for adolescent obesity (35). Mechanistically, research investigating the adverse effects of late bedtime on weight development found that insufficient sleep duration caused by delayed bedtime may lead to postponed mealtimes (mean delay: 35 min, 95% CI: 16–53, *p* < 0.001) and increased caloric intake (β = 131.51, 95% CI: 87.47–175.56, Z = 5.85, *p* < 0.001) (36). These findings underscore the potential negative impact of bedtime on childhood weight outcomes, emphasizing the necessity of addressing both sleep duration and sleep timing in obesity prevention strategies.

To further analyze and visually present the relationship between sleep duration, bedtime, and weight development, this study employed RCS models, combined with logistic regression, to explore the non-linear associations among variables. Compared with the reference point of 10.5 h defined in this study, children with shorter sleep duration exhibited a higher risk of adverse outcomes. This finding is consistent with the study by Tuerxun et al., which identified a significant statistical correlation between shorter sleep patterns and both general and abdominal obesity among children aged 3–6 years in Wuhan (16). Similarly, a cohort study on the development of Japanese children demonstrated that children with shorter sleep duration at 2.5 years old had a higher risk of obesity at 5.5 years old, with a more pronounced effect as sleep duration progressively decreased, indicating a trend association (37). In contrast, in the RCS model results for bedtime, fluctuations in the odds ratio were observed. Therefore, in this study, participants were stratified based on bedtime, and an additive interaction model was applied to comprehensively analyze the combined effects of sleep duration and bedtime, as well as the interaction between sleep duration and sleep quality, on childhood overweight and obesity.

Existing studies predominantly utilize standardized instruments such as the Brief Infant Sleep Questionnaire (BISQ-E) (38) and the CSHQ (39) to assess pediatric sleep quality. In this study, sleep quality was evaluated by using the CSHQ. Additive interaction model analyses demonstrated that children with sleep problems but maintaining moderate (9–11 h) or extended (>11 h) sleep durations exhibited protective associations against overweight or obesity compared with those with optimal sleep quality (OR = 0.814, 95% CI: 0.733–0.905; OR = 0.759, 95% CI: 0.678–0.857, respectively). This paradoxical association may reflect compensatory mechanisms, whereby extended sleep duration in children categorized with poor sleep quality offsets its detrimental impacts.

Internationally recognized guidelines from the World Health Organization (WHO), the National Sleep Foundation, and the American Academy of Pediatrics recommend age-specific sleep durations (preschoolers: 10–13 h; school-aged children: 9–11 h; adolescents: 8–10 h) (40) and bedtimes (preschoolers: 19:00–21:00; school-aged children: 19:00–22:00; adolescents: 20:00–23:00) (24). Using children meeting these recommendations as the reference group, adjusted analyses revealed distinct risk patterns: late bedtime (after 22:00) elevated overweight or obesity risk among children with moderate sleep duration (OR = 1.214, 95% CI: 1.069–1.379), whereas early bedtime (before 21:00) reduced risk (OR = 0.853, 95% CI: 0.765–0.951). Insufficient sleep duration (<9 h) consistently increased risk regardless of bedtime. Notably, extended sleep duration (>11 h) with early bedtime retained protective effects against abnormal weight outcomes, yet failed to mitigate risks associated with late bedtime (OR = 0.983, 95% CI: 0.695–1.392).

Despite these novel findings, several limitations should be acknowledged. First, the questionnaires in this study primarily focused on children’s sleep and growth parameters, omitting parental BMI data, which may lead to residual confounding by unmeasured genetic factors. Meanwhile, the data rely on parental reports, which are inherently subject to recall bias. Second, the cross-sectional design with single time-point data precludes causal inference, providing only evidence of statistical associations. To address these limitations, our team is establishing the Hainan Island Birth Cohort (HIBC) to longitudinally validate potential causal relationships.

## Conclusion

This population-based study of 19,047 children in southern China, focusing on multidimensional sleep patterns (duration, quality, and timing), reveals the following three pivotal insights into pediatric overweight or obesity:

**Sleep duration and bedtime synergy**: Short sleep duration (<9 h) and late bedtime (after 22:00) independently and synergistically elevate obesity risk (OR = 1.186 and 1.121, respectively), while adherence to age-specific sleep guidelines (9–11 h with bedtime before 21:00) confers protection (OR = 0.853, 95% CI: 0.765–0.951).

**Non-linear dose–response**: Restricted cubic spline analyses revealed a non-linear relationship, where obesity risk escalates markedly below 9 h of sleep, despite partial risk attenuation with extended sleep (>11 h).

**Sleep quality paradox**: Suboptimal sleep quality paradoxically associates with reduced obesity risk (OR = 0.825, 95% CI: 0.759–0.889), likely mediated by compensatory prolonged sleep duration offsetting quality deficits.

These findings underscore sleep duration and bedtime as modifiable targets for obesity prevention, while highlighting the complexity of the role of sleep quality’ in weight regulation.

## Data Availability

The raw data supporting the conclusions of this article will be made available by the authors without undue reservation.
